# Pax3-induced expansion enables the genetic correction of dystrophic satellite cells

**DOI:** 10.1186/s13395-015-0061-7

**Published:** 2015-10-26

**Authors:** Antonio Filareto, Fabrizio Rinaldi, Robert W. Arpke, Radbod Darabi, Joseph J. Belanto, Erik A. Toso, Auston Z. Miller, James M. Ervasti, R. Scott McIvor, Michael Kyba, Rita CR Perlingeiro

**Affiliations:** Department of Medicine, Lillehei Heart Institute, University of Minnesota, 4-128 CCRB, 2231 6th St. SE, Minneapolis, MN 55455 USA; Department of Pediatrics, Lillehei Heart Institute, University of Minnesota, Minneapolis, MN 55455 USA; Department of Biochemistry, Molecular Biology, and Biophysics, University of Minnesota, Minneapolis, MN 55455 USA; Department of Genetics, Cell Biology and Development, University of Minnesota, Minneapolis, MN 55455 USA

**Keywords:** Satellite cells, Muscular dystrophy, Gene correction, Sleeping Beauty, Dystrophin, Pax3, Regeneration

## Abstract

**Background:**

Satellite cells (SCs) are indispensable for muscle regeneration and repair; however, due to low frequency in primary muscle and loss of engraftment potential after ex vivo expansion, their use in cell therapy is currently unfeasible. To date, an alternative to this limitation has been the transplantation of SC-derived myogenic progenitor cells (MPCs), although these do not hold the same attractive properties of stem cells, such as self-renewal and long-term regenerative potential.

**Methods:**

We develop a method to expand wild-type and dystrophic fresh isolated satellite cells using transient expression of Pax3. This approach can be combined with genetic correction of dystrophic satellite cells and utilized to promote muscle regeneration when transplanted into dystrophic mice.

**Results:**

Here, we show that SCs from wild-type and dystrophic mice can be expanded in culture through transient expression of Pax3, and these expanded activated SCs can regenerate the muscle. We test this approach in a gene therapy model by correcting dystrophic SCs from a mouse lacking dystrophin using a *Sleeping Beauty* transposon carrying the human *μDYSTROPHIN* gene. Transplantation of these expanded corrected cells into immune-deficient, dystrophin-deficient mice generated large numbers of dystrophin-expressing myofibers and improved contractile strength. Importantly, in vitro expanded SCs engrafted the SC compartment and could regenerate muscle after secondary injury.

**Conclusion:**

These results demonstrate that Pax3 is able to promote the ex vivo expansion of SCs while maintaining their stem cell regenerative properties.

**Electronic supplementary material:**

The online version of this article (doi:10.1186/s13395-015-0061-7) contains supplementary material, which is available to authorized users.

## Background

Duchenne muscular dystrophy is a fatal neuromuscular disease affecting about 1 in 5000 boys [1], which is caused by mutations in the gene coding for the dystrophin protein [2]. Its absence results in continual damage and regeneration, which becomes impaired over time, leading to displacement of muscle fibers with fat and connective tissue, resulting in diminished muscle function [3]. Loss of regeneration is thought to be due to exhaustion or impairment of satellite cells. These are resident adult muscle stem cells located underneath the basal lamina [4] that actively contribute to skeletal muscle growth and regeneration throughout life [5]. Under normal physiological conditions, satellite cells are quiescent [6] and express Pax7 [7]. In response to injury, satellite cells become activated and proliferate as myogenic progenitor cells (MPCs), migrate to the damaged area, and fuse to form new multinucleated myofibers [8, 9]. This period of extensive proliferation terminates when homeostasis is achieved and injury is repaired [10]. During this process, a small proportion of satellite cells re-enter quiescence [11, 12] and reconstitute the satellite cell pool. These characteristics make satellite cells an attractive cell population to be utilized in cell-based therapies to treat muscular dystrophies.

Numerous studies have investigated the regenerative potential of satellite cells and their progeny derivatives [10, 13–20]. However, ex vivo expanded satellite cells have diminished regenerative potential in vivo [21]. Tremblay and colleagues have conducted a phase I clinical trial for MPC cultures using a high-density injection approach in DMD patients, which has been reported encouraging in the sense dystrophin expression could be detected [22–24]. Nevertheless, MPCs are not the most desirable population to be transplanted since these cells are characterized by limited survival and migration upon injection [25–28] and, more importantly, are devoid of self-renewal, which limits their long-term regeneration potential. Thus, an essential requirement for muscle cell-based therapies is the development of an approach that enables the ex vivo expansion of satellite cells while maintaining their “stemness” and regeneration potential.

Here, we show that this can be accomplished in mouse satellite cells by transient expression of Pax3, the master regulator of the embryonic myogenic program, and that ex vivo expanded satellite cell progeny have a high capacity for engraftment and regeneration. Most importantly, we demonstrate that this system can be combined with genetic correction of cells from dystrophic animals and utilized to promote muscle regeneration and improve functional properties in vivo when transplanted back into dystrophic mice.

## Methods

### Mice and satellite cell isolation

Animal maintenance and experimental use were performed according to protocols approved by the University of Minnesota Institutional Animal Care and Use Committee. The transgenic Pax7-ZsGreen reporter mouse line was generated by injection of the purified Pax7-BAC (RP23–218H13) containing a ZsGreen fluorescence protein into the first coding exon, as described [29]. Pax7-ZsGreen/*mdx* mice were generated by breeding *mdx* mice (C57BL/10ScSn), purchased from Jackson Laboratories (Bar Harbor, ME, http://www.jax.org), to WT-Pax7-ZsGreen mice [29]. Female progeny containing both genes were crossed to hemizygous *mdx* male mice. R26-M2rtTA/M2rtTA mice [30] were also bred to Pax7-ZsGreen mice. Resulting mice from this breeding were intercrossed, and mice homozygous for at the R26-M2rtTA were identified. *NSG-mdx*^*4Cv*^ mice [31] were used as transplantation recipients. Pax7-ZsGreen satellite cells were isolated from *soleus* (SOL), *extensor digitorum longus* (EDL), *tibialis anterior* (TA), and *gastrocnemius* (GAS) muscles of 6–8-week-old Pax7-ZsGreen/mdx or R26-M2rtTA/M2rtTA;Pax7-ZsGreen mice, as described previously [29]. Analysis and cell sorting were performed on a Cytomation MoFlo cytometer (Dako, Carpinteria, CA, http://www.dako.com).

### Generation of Pax3-induced cells

Freshly isolated satellite cells were immediately transduced with the inducible Pax3-IRES-mCherry-expressing lentivector [32] to generate the Pax3-induced satellite cells and *mdx*-Pax3-induced satellite cells; control satellite cells were transduced with mCherry lentiviral vector.

### Sleeping Beauty system and generation of corrected *μDYS*-Pax3-induced cells

We developed a bicistronic T2-inverted terminal repeat transposon (Tn) vector (pKt2-GFP-Neo/μ*DYSTROPHIN*) carrying an 11.3-kb engineered transgene containing the skeletal α-actin promoter (pHSA) (generously provided by Jeffrey Chamberlain, Department of Neurology, University of Washington School of Medicine) that drives *μDYS* and a ubiquitin promoter (hEF1a-eIF4g) that drives a GFP-2A-Neo reporter gene, which allows for the selection of *μDYS*-corrected *mdx*-Pax3-induced cells. The whole transgene is flanked by the terminal inverted repeats (IR/DR), each of which contains two binding sites for the transposase. For the transposase, we used pCMV-SB100X (generously provided by Zoltan Ivics from Max Delbruck Center for Molecular Medicine, Berlin), which yields high levels of Tn integration. SB transposase-mediated gene delivery was done using an Amaxa Nucleofector (Lonza) according to the manufacturer’s protocol (fibroblast Nucleofector kit solution, Nucleofector program U-023). 5 × 10^5^*mdx*-Pax3-induced cells were nucleofected with 1 μg of pCMV-SB100X and 4 μg of Tn:pKt2-GFP-Neo/μH2-*μDYS*. Corrected *μDYS*-Pax3-induced satellite cell (SC) Pax3 cells were purified based on sorting for GFP^+^ cells. The *μDYS* was generated using the full-length human dystrophin cDNA in the Gateway entry vector pENTR223.1 (NM_004006) that was obtained from the ORFeome Collaboration. The entry vector was N-terminally FLAG-tagged via PCR using primers with overhangs encoding the tag. The μ -dystrophin^ΔR4–23/ΔCT^ (*μDYS*) was built by deletion using previously described methods [33]. Briefly, PCR primers were designed such that they amplified the entire plasmid except the region being deleted, namely spectrin-like repeats 4–23. These linear PCR products were then circularized via the addition of T4 polynucleotide kinase and T4 DNA ligase (New England Biolabs) and sequence verified. A second round of PCR and circularization was performed to delete the C-terminus. All PCRs were performed using PfuUltra II HS polymerase (Stratagene).

### RNA extraction and real-time PCR analysis

RNA was isolated from cultured cells using TRIzol reagent (Life Technology). One microgram of total RNA was reverse-transcribed using the ThermoScript™ Reverse Transcriptase kit (Life Technology). In control uninduced and Pax3-induced cells, real-time PCR was performed for muscle-specific genes with probe sets from Applied Biosystems [34]. To confirm *μDYS* expression in corrected Pax3-induced cells, specific primers were designed for the *μDYS* gene (F: 5′-TTCTAAGTTTGGGAAGCAGCA-3′ and R: GGTCTGGCCTATGACTATGGA. Primers for GAPDH were F: AGGCCGGTGCTGAGTATGTC and R: TGCCCTGCTTCACCACCTTCT).

### Muscle injury and transplantation studies

Four-month-old *NSG-mdx*^*4Cv*^ mice were used as recipients for all transplantation studies described here. Muscle injury was performed as described previously [31]. Briefly, both hind limbs were subjected to 1200 cGy of irradiation at day 2; muscle injury was induced 24 hours later (day 1) using 15 μl of cardiotoxin (10 μM, SIGMA) in both right and left TA muscle; on day 0, cells were injected into the left TA of each mouse using a Hamilton syringe. For each set of transplantation, cells were collected using cell dissociation buffer, enzyme-free (GIBCO) (10 min at 37 °C), resuspended in PBS, and then injected directly into the left TA muscle (350,000 cells per 10 μl PBS). Control TA muscles were injected with the same volume of PBS.

### Immunofluorescence of cultured cells and tissue sections

TA muscles were embedded in Tissue-Tek OCT compound and immediately frozen in liquid nitrogen-cooled isopentane. Cut tissues (10–12 μm) were permeabilized with 0.3 % Triton X-100 in PBS for 10 min, then blocked for 1 h in 20 % goat serum, and incubated overnight with specific primary antibody in antibody diluent (Dako). Primary antibodies used were rabbit anti-dystrophin polyclonal antibody (1:250, ab 15277; Abcam), mouse anti-dystrophin polyclonal antibody specific for human μDys (1:50, MAB1690; Chemicon, Millipore), mouse anti-Pax7 (1:250; MAB 1675; R&D System), rabbit anti-laminin (1:400; Sigma), anti-rabbit ZsGreen (1:100; Clontech), and anti-embryonic MHC (1:20; F1.652; Developmental Studies Hybridoma Bank). For ZsGreen staining, tissues were collected and immediately fixed in 4 % PFA for 1 h. Next slides were incubated in a solution of 30 % sucrose in 0.01 M PBS for 2 h and left over night in a solution of 20 % sucrose in 0.01 M PBS. The next day, TA muscles were embedded in OCT compound (Leica). A MOM kit (Vector Laboratory) was used following the manufacturer’s instruction. After three PBS washes, sections were incubated for 45 min with secondary antibody. For secondary staining, goat Alexa-555 anti-rabbit or mouse, Alexa-488 anti-rabbit or mouse, Alexa-647 anti-rabbit, and Alexa-488 anti-chicken (1:1000) were used (Molecular Probes). Control tissues were processed simultaneously in the same manner.

For in vitro cultures, cells were maintained on gelatin-coated plates and processed as described above. Cells were first fixed for 10 min at RT in 4 % PFA, washed twice in PBS, and incubated for 10 min with 0.3 % Triton X-100 in PBS. The following primary antibodies were used: anti-Pax3 (1:100; R&D Systems) and anti-MHC (1:50; MF20; Developmental Studies Hybridoma Bank). Alexa Fluor 555 goat anti-rabbit and anti-mouse (Molecular Probes) was used for secondary staining. 4, 6-Diamidino-2-phenylindole (DAPI) was used to counter-stain nuclei (Sigma).

### Muscle preparation for mechanical studies

For the measurement of contractile properties, mice were anesthetized with avertin (250 mg kg^−1^ intraperitoneal) and analyzed as described previously [28, 30]. Intact TA muscles were analyzed ex vivo in an experimental organ bath filled with mammalian Ringer buffer, containing platinum electrodes placed longitudinally on either side of the muscle. Muscles were stimulated by electric field (square wave pulses 25 V, 0.2 ms in duration, 150 Hz) using an optimal muscle length (*L*_0_) for the development of maximum isometric tetanic force (*F*_0_). Specific force (s*F*_0_) was determined by normalizing maximum isometric tetanic force (*F*_0_) to cross-sectional area (CSA). Total muscle CSA was calculated by dividing muscle mass (mg) by the product of muscle length (mm), and 1.06 mg/mm^3^ is the density of mammalian skeletal muscle.

## Results and discussion

### Derivation and ex vivo expansion of satellite cells using Pax3

**Fig. 1:**
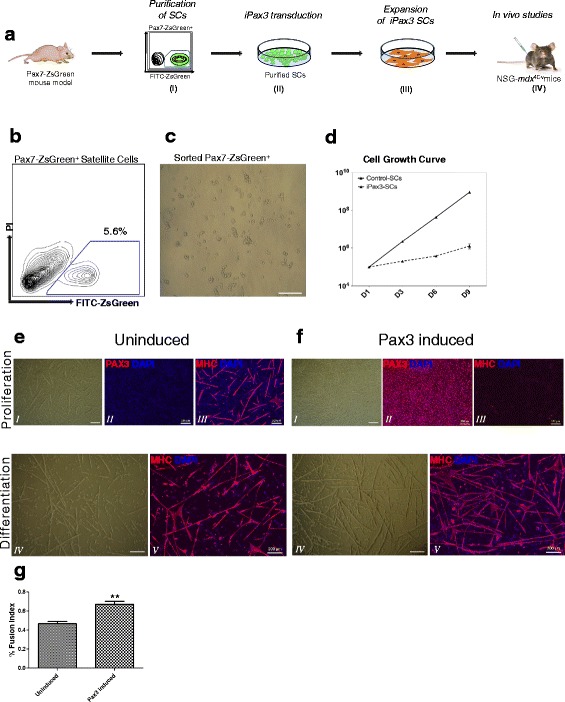
Derivation and characterization of Pax3-induced satellite cells. **a** (*I*) FACS purification of satellite cells based on ZsGreen expression (Pax7), (*II*) transduction of Pax7^+^ cells with an inducible expression system encoding Pax3, (*III*) in vitro expansion of Pax3-induced cells and control empty vector counterparts, and (*IV*) transplantation of iPax3 and control cells into *NSG-mdx* ^*4Cv*^. **b** Representative FACS profile for ZsGreen (Pax7) expression in digested muscles isolated from Pax7-ZsGreen reporter mice. Sorting gate for ZsGreen^+^ (Pax7^+^) satellite cells is shown. **c** Phase-contrast image of sorted ZsGreen^+^ (Pax7^+^) satellite cells. **d** Cell growth curve of Pax3-induced cells and control counterparts at several passages (P1–P4) (*n* = 2, mean ± SD). **e**, **f** In vitro characterization of ex vivo expanded satellite cells grown under proliferation and differentiation culture conditions. Phase-contrast images of control empty vector (**e**) and Pax3-induced (**f**) monolayers. Representative immunofluorescence staining for Pax3 (*red*, *upper panels*) and MHC (*red*, *lower panels*) in control empty vector SCs (**e**) and Pax3-induced SCs (**f**). Cells are co-stained with DAPI (*blue*). *Scale bar* 200 μm. **g** Fusion index calculation. *Error bars* represent s.e.m. (*n* = 3). **P < 0.01

To determine whether SCs maintained engraftment potential when expanded ex vivo using conditional expression of Pax3, we followed the strategy summarized in Fig. 1a, in which SCs from the transgenic Pax7-ZsGreen reporter mouse [27] were (I) purified by flow cytometry, (II) genetically modified with a lentiviral vector encoding a doxycycline-inducible Pax3 transgene, (III) expanded ex vivo in the presence of doxycycline, and then (IV) transplanted into immune-deficient, dystrophin-deficient *NSG-mdx*^*4Cv*^ [31] mice. After enzymatic digestion, the muscle mononuclear fraction of Pax7-ZsGreen was FACS-purified based on ZsGreen expression, which reflects Pax7^+^ cells (Fig. 1b), and accordingly gave rise to a homogeneous SC population (Fig. 1c). These cells were immediately transduced with a doxycycline-regulated conditional Pax3-IRES-mCherry-expressing lentivector (Pax3 induced) [32]. As a control, SCs were transduced with empty vector (mCherry only). Pax3^+^ (mCherry^+^) cells were detected only when doxycycline (dox) was added to the culture medium (Additional file 1). To determine the effect of Pax3 on the expansion of transduced SCs, we evaluated the proliferation rate of Pax3-induced cells side-by-side with control cell preparations (empty vector) grown under identical culture conditions: proliferation medium with basic fibroblast growth factor (bFGF) and dox. Notable expansion advantage was observed in Pax3-induced cultures when compared to control counterparts (Fig. 1d). Although under these proliferation conditions, both control and Pax3-induced cells displayed similar morphology (Fig. 1e, f, panel *I*), only Pax3-induced cells showed abundant Pax3 expression, as evidenced by immunofluorescence staining (Fig. 1e, f, panel *II*) and gene expression analyses (Additional file 2). As expected, Pax3 overexpression in SCs was accompanied by upregulation of its target gene Myf5 [35] (Additional file 2). Under proliferation conditions, Pax3-induced cells showed no signs of myotube formation, as indicated by the absence of signal for myosin heavy chain (MHC) (Fig. 1f, panel *III*, and Additional file 2), whereas the control uninduced population spontaneously differentiated into MHC-positive myotubes (Fig. 1e, panel *III*, and Additional file 2). Nevertheless, when Pax3-induced and control cells were subjected to differentiation conditions (5 % horse serum and withdrawal of dox and bFGF), both cultures gave rise to multinucleated myotubes displaying abundant expression of MHC (Fig. 1e, f, panels *IV* and *V*, and Additional file 2). Control cultures expressed significant levels of MHC under proliferation conditions, suggesting the propensity of these cells to begin differentiation as soon as they have reached confluence. We next quantified the fusion index of control and Pax3-induced cultures. Upon in vitro differentiation, Pax3-induced SCs exhibited elevated fusion index (67 %) relative to control cultures (47 %). Thus, under the conditions tested here, Pax3 induction allows the in vitro expansion of less differentiated SCs, without affecting their ability to terminally differentiate into fusing myotubes.

### In vivo regenerative potential of ex vivo expanded satellite cells

**Fig. 2:**
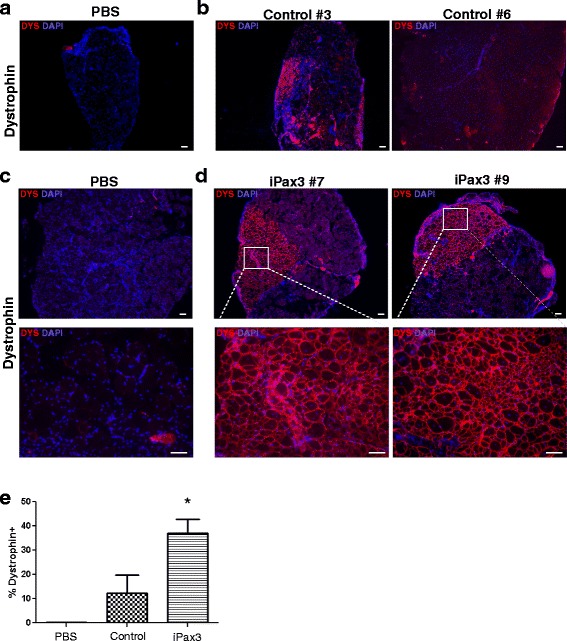
Regenerative potential of Pax3-induced satellite cells following their transplantation into *NSG-mdx* ^*4Cv*^ mice. Engraftment analysis of control empty vector (**a**, **b**) and Pax3-induced cells (iPax3) (**c**, **d**). Cross sections of TA muscles harvested from *NSG-mdx* ^*4Cv*^ mice that had been injected with PBS (**a**, **c**) or satellite cells (**b**, **d**) were stained with antibody to dystrophin (*red*). Engrafted tissues from control and Pax3-induced cells are represented by mice *#03* and *#05* and *#07* and *#09*, respectively. DAPI is shown in *blue. Scale bar*, 50 μm. **e** Quantification of DYS^+^ myofibers in treated muscles. *Error bars* represent s.e.m. (*n* = 6). *P < 0.03

To evaluate in vivo repopulation potential after 1 week of ex vivo expansion, Pax3-induced and respective control cell preparations were transplanted into the TA muscles of *NSG-mdx*^*4Cv*^ mice. Prior to cell transplantation, both hind limbs were subjected to irradiation (12 Gy/leg) to deplete endogenous SCs [31] and injury with cardiotoxin (CTX). While the contra-lateral TA was injected with PBS, 350,000 Pax3-induced or control cells were injected into the right TA. Five weeks after transplantation, TA muscles were harvested and evaluated for engraftment by immunofluorescence staining for dystrophin. Whereas DYS^+^ myofibers were virtually undetectable in PBS-injected muscles (Fig. 2a, c), dystrophin expression was observed in TA muscles that had been transplanted with control (Fig. 2b) or Pax3-induced (Fig. 2d) cell preparations, with the latter showing higher engraftment levels (Fig. 2e, 14 ± 7.4 vs. 37 ± 5.7 %, respectively).

**Fig. 3:**
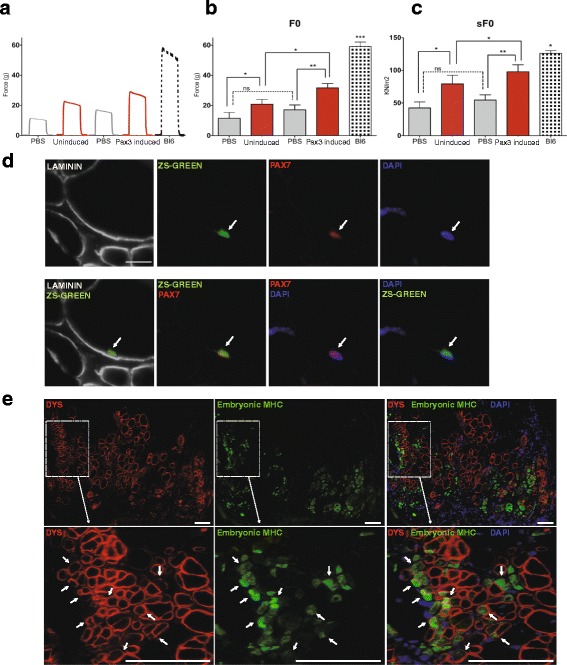
Contractile properties of transplanted muscles and satellite cell homing. **a** Representative examples of maximum isometric tetanic force in TA muscles that had been injected with PBS (contra-lateral leg, *gray line*) and control or Pax3-induced cells (*red lines*). Wild-type Bl6 mice were used for reference control (*dashed line*). **b**, **c** Cell transplantation produces an improvement in absolute (*F*_0_, **b**) and specific (s*F*_0_ = *F*_0_ normalized to CSA, **c**) force. *Error bars* represent s.e.m. from a total of six mice. *P < 0.05, **P < 0.01, ***P < 0.001. **d** In situ analysis reveals the presence of donor-derived satellite cells (ZsGreen/Pax3-induced cells) in the host stem cell pool, as shown by the presence of cells co-stained for both Pax7 (*red*) and ZsGreen (*green*) (*white arrow*) beneath the basal lamina (*gray*). **e** Upon reinjury, engrafted donor-derived satellite cells give rise to newly formed myofibers, as indicated by the co-expression of DYS (*red*) and embryonic MHC (*green*) (*white arrow*). *Arrowheads* denote DYS^−^/eMHC^+^ host-derived new formed myofibers. DAPI is shown in *blue. Scale bar*, 50 μm

Next, we determined whether myofiber engraftment was accompanied by improvement in muscle strength. As expected, the maximum isometric force for PBS-injected TA muscles (contra-lateral legs) was low (Fig. 3a, gray lines). In contrast, engrafted TA muscles showed enhanced isometric force (Fig. 3a, red lines). Cell transplantation of both control and Pax3-induced preparations resulted in improved absolute (Fig. 3b) and specific (Fig. 3c) force of engrafted muscles when compared with their respective PBS-injected contra-lateral muscles. However, muscles that had been transplanted with Pax3-induced cells displayed significantly superior functional improvement (Fig. 3b, c) when compared to control cells (1.52-fold). No statistical difference was observed in forces between the contra-lateral legs (PBS) of the two groups of mice. These results demonstrate that 7-day cultured SCs expanded with Pax3 have a superior ability to improve muscle function, compared to control empty vector transduced counterparts.

To assess whether Pax3-induced cells have the capacity to engraft the host SC compartment, and therefore contribute to ongoing regeneration, engrafted TA muscles were stained for ZsGreen and Pax7 to identify donor-derived SC contribution. Histological analysis of transverse sections of TA muscles 1 month after transplantation clearly identified the presence of Pax7^+^ZsGreen^+^ cells beneath the basal lamina, suggesting that Pax3-induced cells can engraft the SC pool (Fig. 3d). To investigate whether donor-derived iPax3 SCs would be able to contribute to ongoing muscle regeneration, a cohort of mice transplanted with unlabelled Pax3-induced cells were reinjured with CTX 1 month after cell transplantation. Ten days after reinjury, we detected donor-derived newly regenerated myofibers, as indicated by the presence of DYS^+^/embryonic MHC^+^ myofibers (Fig. 3e, white arrows). Since we have used half of the usual dose of CTX (5ul/5uM, instead of 10ul/10uM) for these reinjury studies, CTX injection did not result in degeneration of the whole tissue, and accordingly the presence of DYS^+^/eMHC^−^ fibers was detected. These results suggest that at least some of engrafted Pax3-induced cells remain less differentiated and are able to respond to a second round of muscle injury.

### Genetic repair of dystrophic Pax3-induced cells

**Fig. 4:**
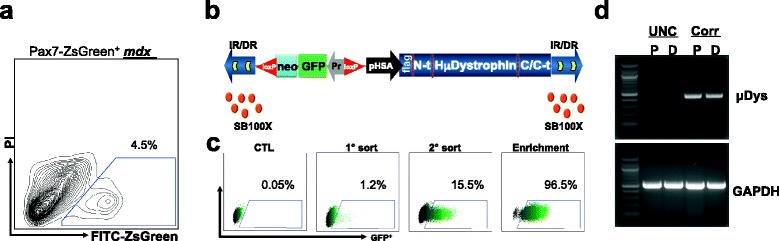
Correction of dystrophin-deficient Pax3-induced satellite cells using a human *μDYS* transgene. **a** FACS plot shows gate for the purification of ZsGreen^+^ (Pax7^+^) satellite cells from Pax7-ZsGreen**/** *mdx* mice. **b** The *Sleeping Beauty transposon* system consists of transposon (Tn) and transposase (SB100X) vectors. The Tn is a bicistronic promoter vector of 11.3 Kb containing the ubiquitin hEF1a-eIF4g (Pr, in *gray*) and the skeletal muscle-specific skeletal α-actin promoter (pHSA, in *black*). The ubiquitin promoter drives a GFP-2A-Neo. This selection marker cassette is flanked by lox P sequences (*red*). The human *μDYS* gene is under control of the pHSA. SB100X transposase proteins (*red spheres*) bind the DR sequences (*yellow arrows*) within the two inverted repeats (IR/DR, *arrowheads*) and catalyze integration of the whole transposon transgene into the genome with high efficiency. **c** Representative FACS profiles for enrichment steps used to isolate a pure and stable population of corrected GFP^+^ cells (*μDYS*-Pax3-induced cells) following transfection with pKT2/*μDYS* and SB100X. Control consisted of dystrophin-deficient Pax3-induced cells (*CTL*) nucleofected with pKT2 transposon vector only (no transposase). **d** RT-PCR analysis for uncorrected (*UNC*, dystrophin-deficient Pax3-induced cells) and corrected (*Corr*, *μDYS*-Pax3-induced cells) cells grown under proliferation (*P*) and differentiation (*D*) culture conditions shows the expression of human *μDYS* solely in corrected cells. GAPDH was used as housekeeping gene

We next applied genetic correction to ex vivo expanded dystrophic SCs following the protocol outlined in Fig. 1a, but using SCs harvested from *mdx* mice bred to carry the Pax7-ZsGreen reporter (Fig. 4a). For genetic repair, we used the human micro-dystrophin^ΔR4–23/ΔCT^ (*μDYS*) transgene lacking the spectrin-like repeats 4–23 and the C-terminus [36] and the non-viral *Sleeping Beauty system* for transduction. First, we generated a Tn vector (pKT2-Neo selection marker driven by the-Neo/hH2 *μDYS*; Fig. 4b) containing two divergent genes: a GFP/Neo selection marker driven by the hEF1a-eIF4g promoter and the human μDystrophin (*μDYS*) gene under the control of a pHSA [37].

SCs were isolated by flow cytometry from Pax7-ZsGreen**/***mdx* mice (Fig. 4a), immediately transduced with the doxycycline-inducible Pax3 vector, and grown in doxycycline to induce Pax3 expression. It should be noted that almost immediately upon placing the Pax7-ZsGreen SCs into culture, the ZsGreen fluorescence is lost. We now then transduced these non-fluorescent cells with the μ-dystrophin correction vector, which contained a GFP reporter, and sorted on this signal; therefore, the culture was now constitutively green. Dystrophin-deficient Pax3-induced cells were subsequently nucleofected with Tn vector and transposase (engineered hyperactive variant SB100X [38]; Fig. 4b, upper panel), using a plasmid ratio of 4:1, respectively, which we have previously found to provide optimal in vitro gene transfer for a large transgene [32]. Five days after nucleofection, flow cytometry analysis revealed a cell sub-population positive for GFP/*μDYS* (~1.2 %) (Fig. 4b, lower panel). Following two rounds of sorting, a highly enriched *μDYS*^+^ (GFP^+^) population was obtained (>96 %) (Fig. 4b, lower panel). Expression of the transgene in corrected cells was confirmed by RT-PCR analysis using specific primers for the human *μDYS* transgene (Fig. 4c). These results demonstrate the capacity for the *Sleeping Beauty* system to deliver a large transgene (11.3 Kb) into dystrophic activated SCs.

### Regenerative potential of *μDYS*-Pax3-induced cells

**Fig. 5:**
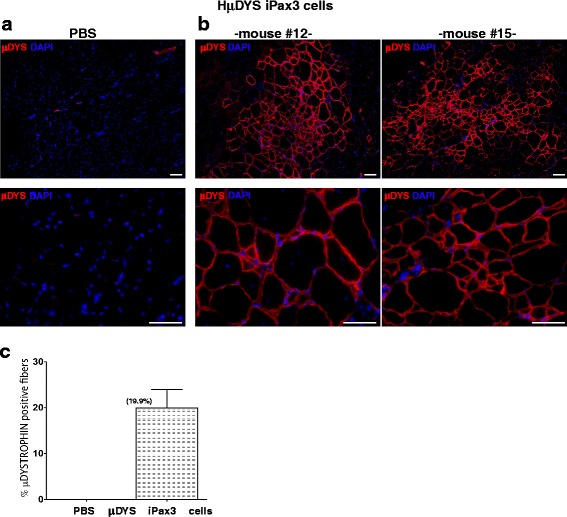
Engraftment of *μDYS*-Pax3-induced cells into *NSG-mdx* ^*4Cv*^ mice. TA muscles harvested from *NSG-mdx* ^*4Cv*^ mice that had been injected with PBS (**a**) or corrected ex vivo expanded satellite cells (*μDYS*-Pax3-induced cells) (**b**) were stained using an antibody specific for human DYSTROPHIN (*red*). The DYS protein was detected only in the transplanted muscles. Two representative transplanted mice (**b**) are shown. DAPI is shown in *blue. Scale bar*, 50 μm. **c** Quantification of human μDYSTROPHIN^+^ myofibers in these transplanted muscles. *Error bars* represent s.e.m (*n* = 6)

To assess the regenerative potential of corrected μDys-Pax3-induced cells in vivo, these cells were transplanted into CTX-injured TA muscles of *NSG-mdx*^*4Cv*^ mice. We did not irradiate these mice as irradiation would be discouraged in the clinical setting. One month following transplantation, TA muscles were harvested and sections were evaluated for engraftment by immunostaining using a human DYSTROPHIN antibody that recognizes the N-terminal epitope, which is preserved in the human *μDYS* transgene. While no DYS expression was detected in PBS-injected muscles (Fig. 5a), muscles that had been transplanted with *μDYS*-Pax3-induced cells generated large engrafted areas with DYS^+^ myofibers (Fig. 5b). Quantification of engraftment revealed that approximately 20 % of fibers in transplanted muscles were μDYSTROPHIN^+^, confirming the regeneration potential of ex vivo corrected activated SCs.

**Fig. 6:**
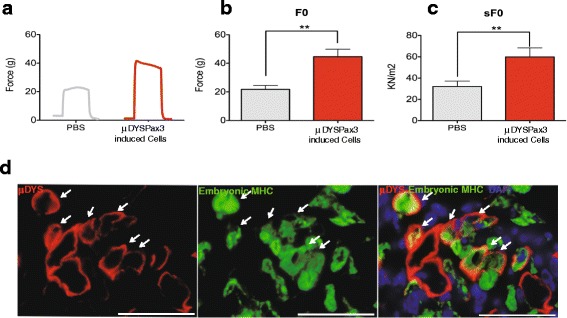
Contractile function and response to reinjury by muscles engrafted with *μDYS*-Pax3-induced cells. **a** Representative examples of maximum isometric tetanic force in TA muscles that had been injected with PBS (contra-lateral leg, *gray line*) or Pax3 induced (*red line*). **b**, **c** *μDYS*-Pax3-induced cell transplantation produced a significant improvement in absolute (*F*_0_, **b**) and specific (s*F*_0_ = *F*_0_ normalized to CSA, **c**) forces. *Error bars* represent s.e.m. from a total of six mice. **P < 0.01. **d** Immunofluorescence staining for embryonic MHC and μDYS in engrafted TA muscles analyzed 10 days after CTX reinjury indicates the presence of newly formed donor myofibers as denoted by co-expression of human μDYS (*red*) and eMHC (*green*) (*arrows*). *Arrowheads* show μDYS^−^/eMHC^+^ host-derived newly formed myofibers. Alexa-647 was used to detect eMHC. DAPI is shown in *blue. Scale bar*, 50 μm

We next investigated whether engraftment of corrected *μDYS*-Pax3-induced cells was accompanied by functional improvement. Engrafted muscles showed superior isometric (Fig. 6a), absolute (Fig. 6b), and specific (Fig. 6c) force when compared to PBS-injected TA muscles.

To determine whether engrafted *μDYS*-corrected Pax3-induced cells would have the same ability to respond to injury as shown above for WT cells and would therefore be capable of providing μDYSTROPHIN continuously, we reinjured muscles that had been previously transplanted with *μDYS*-Pax3-induced cells. Ten days following CTX injection, we stained muscle sections with embryonic MHC and human DYS antibodies. This clearly showed the presence of donor-derived newly regenerated muscle fibers that were double-positive for μDYS and embryonic MHC (Fig. 6d, white arrows and Additional file 3). Altogether, these results show that transplantation of corrected *μDYS*-Pax3-induced cells provides functional improvement of dystrophic muscles, both in terms of muscle force generation and in terms of their ability to respond to ongoing muscle injury and stably express μDYS protein.

SCs isolated by flow cytometry have been demonstrated to possess a tremendous capacity to improve muscle function in *mdx* mice [31]; however, the impracticality of isolating large numbers of SCs from living donors as well as the requirement for gene correction, if considering an autologous transplantation setting, necessitates ex vivo expansion. To date, only one study has reported a combined cell/gene therapy approach using SCs in the context of muscular dystrophy [18]. In this study, the authors isolated SCs from a dystrophic mouse, transduced them with a lentiviral vector encoding the mouse *μDYS* transgene, and immediately transplanted them into the dystrophic muscle and found that they were able to differentiate into DYS+ fibers.

Several studies have investigated the transplantation of cultures derived from prospectively isolated SCs. Blau and colleagues demonstrated that culturing mouse SCs on a substrate that mimics muscle tissue elasticity, and in the presence of an inhibitor for p38MAPK, helped maintain “stemness” features [10, 39]. Following a different approach, Tapscott and colleagues expanded freshly isolated canine SCs by activating the Notch signaling pathway, which bestowed superior in vivo regenerative ability upon SC-initiated cultures compared to controls [20]. In a recent study, Rudnicki and colleagues reported that short treatment of SCs with Wnt7a resulted in enhanced engraftment that was accompanied by improved muscle function [40].

Herein, we demonstrate that upon conditional expression of Pax3, freshly isolated SCs can be successfully expanded when compared to their cultured empty vector control counterparts (Fig. 1d). Following their intramuscular transplantation into dystrophic mice, Pax3-induced cells display greater regenerative potential than control SCs, and engraftment levels correlated with a significant improvement in muscle strength (Fig. 3a–c). Importantly, we also show that engrafted Pax3-induced cells are capable of seeding the SC pool and responding to a second round of CTX-induced damage by generating newly formed DYS^+^ fibers (Fig. 4d, e). In addition, we show that Pax3-induced dystrophic SCs are amenable to genetic correction. Using a non-viral *Sleeping Beauty* system carrying a human *μDYS* transgene, we corrected SCs from dystrophin-deficient mice and found that these were capable of differentiating into functional muscle fibers in vivo (Fig. 5), increasing force generation capacity of dystrophic muscles (Fig. 6a–c), and producing new myofibers upon CTX reinjury that remain positive for the *μDYS* transgene.

## Conclusions

We describe here a method for the ex vivo expansion of SCs that facilitates genetic correction and importantly allows for the retention of a population with the capacity to regenerate muscle function. Unlike viral approaches that target the myofiber, this approach provides a genetic correction that is persistent and not transient as corrected fibers are eventually lost due to normal muscle turnover. If future studies demonstrate that this approach is similarly efficient to expand human SCs, it may be useful to provide human adult stem cell populations endowed with muscle regeneration potential in vivo.

## Additional files

Additional file 1:
**Generation of inducible-Pax3-Cells. **Representative FACS profile of Pax3 inducible cells grown under proliferation culture conditions. Doxycycline was added to the proliferation medium at 0.75 μg/ml. mCherry+ cells are detected only in Pax3-induced cultures (plus Dox). Additional file 2:
**Real time PCR analyses of proliferating and differentiating cultured satellite cells.** Gene expressionanalysis of control (grey bar) and Pax3 induced cells (black bar). Transcripts are normalized to GAPDH. Error bars represent s.e.m. from replicates of three independent experiments. (PDF 134 kb) Additional file 3:
**Long-term engraftment of** ***μDYS*** **-Pax3-induced cells into** ***NSG-mdx*** ^***4Cv***^ **mice and response to reinjury by muscles engrafted with** ***μDYS*** **-Pax3-induced cells.** (a) The DYS protein was detected only in the transplanted muscles at 2 months after transplantation. (b) Immunofluorescence staining for embryonic MHC (green) and μDYS (red) in engrafted TA muscles (lower magnification). Alexa-647 was used to detect eMHC. DAPI is shown in blue. Scale bar, 50 μm (PDF 2192 kb)

## Abbreviations

bFGF: basic fibroblast growth factor
cGy: centigray
CTX: cardiotoxin
DMD: Duchenne muscular dystrophy
dox: doxycycline
DR: direct repeat
DYS: human DYSTROPHIN
eMHC: embryonic myosin heavy chain
GFP: green fluorescent protein
hEF1a-eIF4g: hybrid promoter of human elongation factor 1 and alpha-human eukaryotic initiation factor 4G
iPax3: inducible Pax3
IR: inverted repeat
MHC: myosin heavy chain
MPCs: muscle progenitors cells
NSG mice: NOD scid gamma mice
pHSA: skeletal α-actin promoter
RT-PCR: reverse transcription polymerase chain reaction
SB100X: Sleeping Beauty
SCs: satellite cells
TA: tibialis anterior
Tn: transposon
μDYS: human micro-dystrophin
