# Supplementary material for: Pax3-induced expansion enables the genetic correction of dystrophic satellite cells
Source: Skelet Muscle. 2015 Oct 26;5:36. doi: 10.1186/s13395-015-0061-7 (PMC4620645; doi:10.1186/s13395-015-0061-7)

Additional File 1

Generation of inducible-Pax3-Cells.
Representative FACS profile of Pax3 inducible cells grown under proliferation culture conditions. Doxycycline was added to the proliferation medium at 0.75 µg/ml. mCherry$^+$ cells are detected only in Pax3-induced cultures (plus Dox).

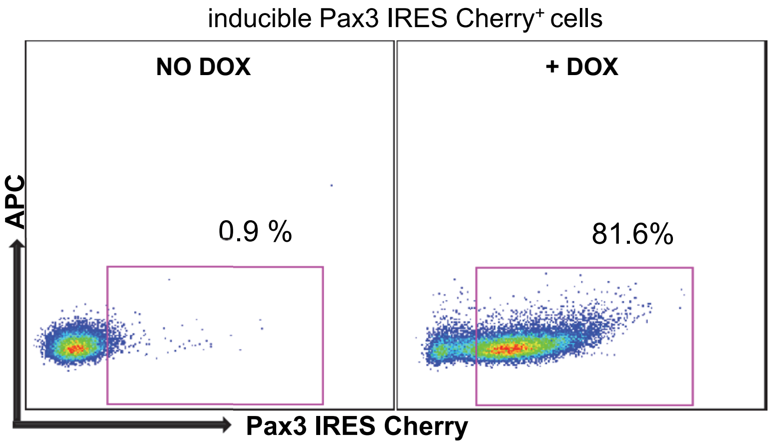

Supplement: Additional file 1: — Generation of inducible-Pax3-Cells. Representative FACS profile of Pax3 inducible cells grown under proliferation culture conditions. Doxycycline was added to the proliferation medium at 0.75 μg/ml. mCherry+ cells are detected only in Pax3-induced cultures (plus Dox). [file 13395_2015_61_MOESM1_ESM.pdf]
