# Supplementary material for: Pax3-induced expansion enables the genetic correction of dystrophic satellite cells
Source: Skelet Muscle. 2015 Oct 26;5:36. doi: 10.1186/s13395-015-0061-7 (PMC4620645; doi:10.1186/s13395-015-0061-7)

Additional File 2

Real time PCR analyses of proliferating and differentiating cultured satellite cells.
Gene expression analysis of control (grey bar) and Pax3 induced cells (black bar). Transcripts are normalized to GAPDH. Error bars represent s.e.m. from replicates of three independent experiments.

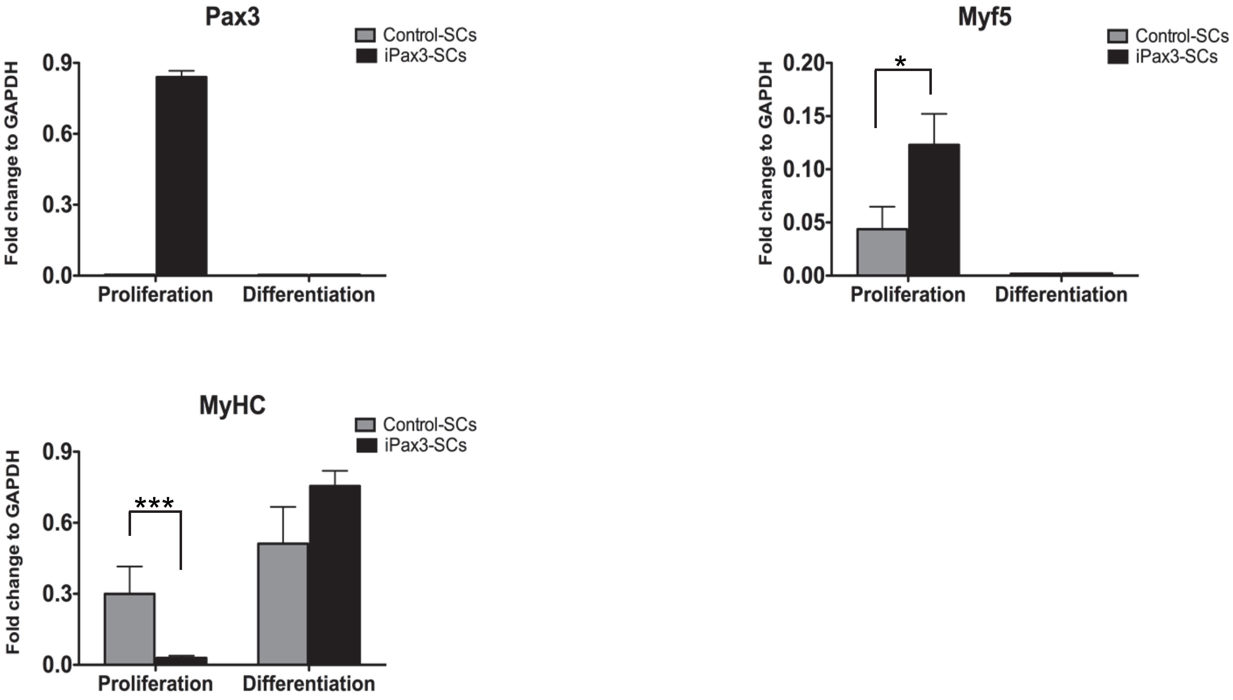

Supplement: Additional file 2: — Real time PCR analyses of proliferating and differentiating cultured satellite cells. Gene expressionanalysis of control (grey bar) and Pax3 induced cells (black bar). Transcripts are normalized to GAPDH. Error bars represent s.e.m. from replicates of three independent experiments. (PDF 134 kb) [file 13395_2015_61_MOESM2_ESM.pdf]
