# Supplementary material for: Pax3-induced expansion enables the genetic correction of dystrophic satellite cells
Source: Skelet Muscle. 2015 Oct 26;5:36. doi: 10.1186/s13395-015-0061-7 (PMC4620645; doi:10.1186/s13395-015-0061-7)

Additional File 3

Long term Engraftment of *μDYS*-Pax3-induced cells into *NSG-mdx^{4Cv}* mice and response to re-injury by muscles engrafted with *μDYS*-Pax3-induced cells. **(a)** The DYS protein was detected only in the transplanted muscles at two months after transplantation. **(b)** Immunofluorescence staining for embryonic MHC (green) and μDYS (red) in engrafted TA muscles (lower magnification). Alexa-647 was used to detect eMHC. DAPI is shown in blue. Scale bar, 50 μm.

a μDYS^{+} myofibers at two months after transplantation

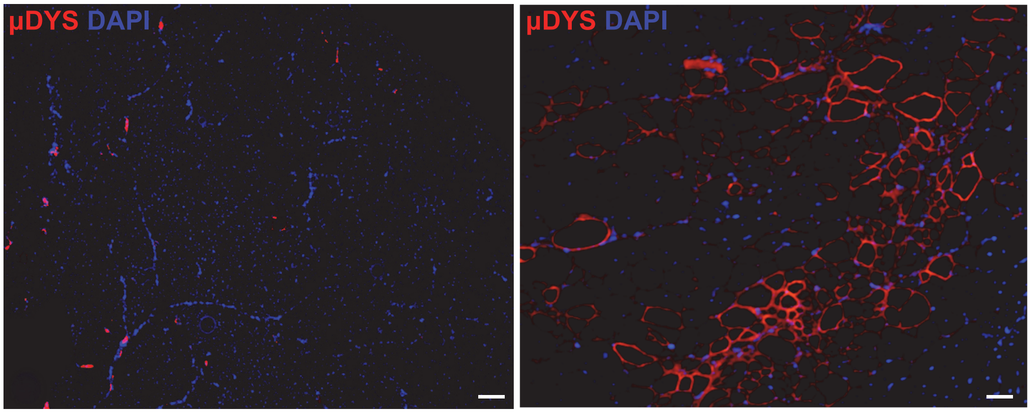

b μDYS^{+} and Embryonic MHC^{+} myofibers after reinjured (lower magnification)

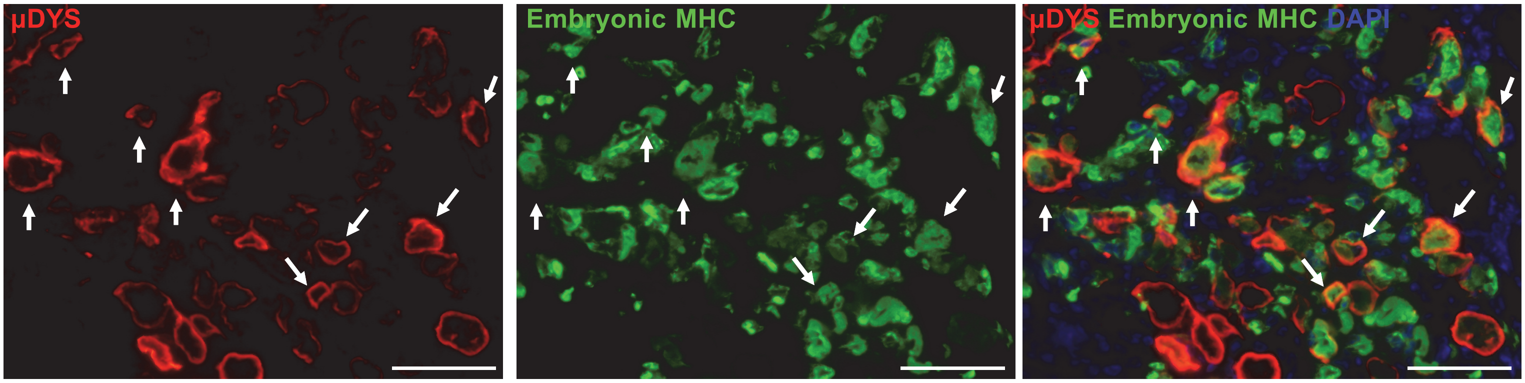

Supplement: Additional file 3: — Long-term engraftment of μDYS -Pax3-induced cells into NSG-mdx 4Cv mice and response to reinjury by muscles engrafted with μDYS -Pax3-induced cells. (a) The DYS protein was detected only in the transplanted muscles at 2 months after transplantation. (b) Immunofluorescence staining for embryonic MHC (green) and μDYS (red) in engrafted TA muscles (lower magnification). Alexa-647 was used to detect eMHC. DAPI is shown in blue. Scale bar, 50 μm (PDF 2192 kb) [file 13395_2015_61_MOESM3_ESM.pdf]
